# EBM BLS: Pitavastatin Reduces Cardiovascular Events in People Living with HIV With Low-to-Moderate Cardiovascular Risk

**DOI:** 10.1007/s11606-026-10258-0

**Published:** 2026-03-09

**Authors:** Suman Atluri, Radha Rao

**Affiliations:** 1 School of Medicine, Baylor College of Medicine, Houston, TX USA; 2 The Margaret M. And Albert B. Alkek Department of Medicine, Baylor College of Medicine, Houston, TX USA

**Keywords:** HIV infection, pitavastatin, cardiovascular disease prevention, primary prevention

**Source Article:** Grinspoon SK, Fitch KV, Zanni MV, et al. Pitavastatin to Prevent Cardiovascular Disease in HIV Infection. N Engl J Med. 2023;389(8):687–699. 10.1056/NEJMoa2304146.

## WHY THIS IS IMORTANT

- People living with HIV (PLWH) are at elevated risk for cardiovascular disease (CVD), now a leading cause of non-HIV-related mortality.^1^
- Guidelines now recommend statin therapy for PLWH aged 40-75 years, regardless of lipid levels or Atherosclerotic Cardiovascular Disease (ASCVD) risk, with the strongest recommendation for those with a 10-year ASCVD risk ≥ 5%. High-intensity statins are advised for risk > 20%, and moderate-intensity statins for lower-risk groups. These recommendations were directly shaped by the REPRIEVE trial.^2^
- Prior to REPRIEVE, 68% of statin-eligible PLWH were not on statins despite meeting criteria.^3^

## INTERVENTION

**Figure 1:**
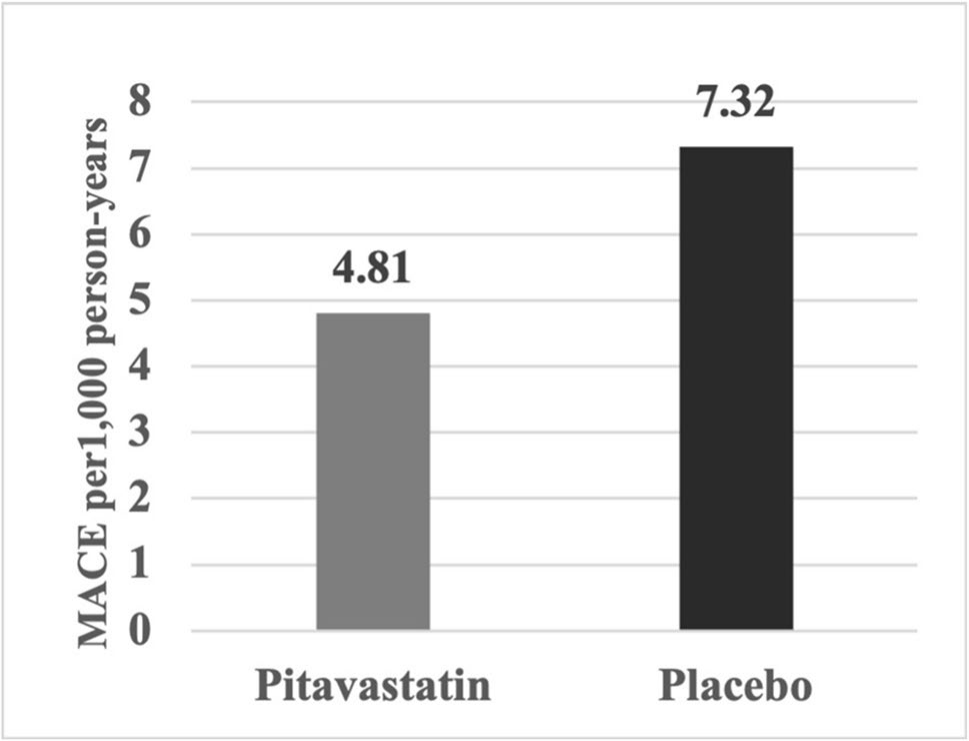
Incidence of MACE over 5.1 years in adults with HIV receiving pitavastatin vs placebo.

- REPRIEVE was a randomized, double-blind, placebo-controlled trial.
- Participants were randomized in 1:1 ratio to pitavastatin (4 mg daily) or placebo.
- Primary Outcome: major adverse cardiovascular events (MACE), defined as the occurrence of any of the following: cardiovascular death, myocardial infarction (MI), hospitalization for unstable angina, stroke, transient ischemic attack (TIA), peripheral arterial ischemia, revascularization of a coronary, carotid, or peripheral artery, or death from an undetermined cause Fig. 1.

## RESULTS

- Median screening CD4 count: 621 cells/mm^3^ with 87.5% having HIV RNA below quantification, confirming viral suppression. Participants had a median age of 50 years; 41.3% were Black, 34.8% White, and 14.6% Asian. All were on antiretroviral therapy, with a median duration of 9.6 years. The median 10-year ASCVD risk was 4.5%.
- Over a median follow-up of 5.1 years, pitavastatin reduced MACE by 35% (4.81 vs. 7.32 events per 1,000 person-years; HR 0.65, 95% CI 0.48–0.90, p = 0.002); trial was stopped early for efficacy.
- Individual MACE components were numerically lower with pitavastatin, although the trial was not powered for these comparisons: MI/cardiac ischemia (1.40 vs 2.51 events per 1,000 person-years), stroke/TIA (1.56 vs 2.36), and cardiovascular mortality (0.64 vs 0.85).
- Muscle-related symptoms (2.3% vs. 1.4%, IRR: 1.74, 95% CI: 1.24—2.45) and incident diabetes mellitus (5.3% vs. 4.0%, IRR: 1.35, 95% CI: 1.09-1.66) were significantly more frequent in the pitavastatin group.

## STUDY DESCRIPTION

### Setting

- Participants were PLWH aged 40–75 years on stable ART with no clinical ASCVD (MI, angina, stroke, TIA, PAD, revascularization), and at low-to-moderate (≤ 15%) 10-year ASCVD risk.
- The study was conducted across 145 sites in 12 countries.

### Exclusion Criteria

- Baseline diabetes with LDL ≥ 70, clinical ASCVD, ASCVD risk > 15%, most active cancer diagnoses, cirrhosis, recent fungal/viral infection, or recent use of statins, PCSK9 inhibitors, or specific immunosuppressants.

### Methods

- Participants received standardized counseling on lifestyle modifications at baseline in addition to pitavastatin or placebo.
- MACE were adjudicated by blinded reviewers.

## STUDY QUALITY AND APPLICATION TO PATIENTS

- The USPSTF quality rating for this study is good.
- Strengths include a diverse population (65.2% non-White, 31.1% women).
- Limitations include few older adults enrolled.
- Traditional risk calculators underestimate ASCVD risk in PLWH: the median estimated 10-year ASCVD risk was 4.5%, yet the observed MACE rate in the placebo group was 7.3%. REPRIEVE used the Pooled Cohort Equations, which—like the newer PREVENT calculator—underestimate risk in this population.^4^
- Guidelines now support statin use for primary CVD prevention in PLWH.
- Pitavastatin was chosen for its minimal drug–drug interactions. Most first-line ART regimens recommended by the U.S. Department of Health and Human Services lack significant statin interactions; notable interactions occur primarily with protease inhibitor–based regimens.
- REPRIEVE enrolled PLWH aged 40–75 on stable ART, most (87%) with suppressed viremia and without ASCVD, liver disease, or recent statin use; thus, findings apply primarily to lower-risk, well-controlled individuals with sufficient life expectancy to realize long-term cardiovascular benefit.

## Data Availability

Data supporting the findings of this study are available in the published source article.
